# Insight into the Mechanism of Salt-Induced Oxidative Stress Tolerance in Soybean by the Application of *Bacillus subtilis*: Coordinated Actions of Osmoregulation, Ion Homeostasis, Antioxidant Defense, and Methylglyoxal Detoxification

**DOI:** 10.3390/antiox11101856

**Published:** 2022-09-20

**Authors:** Mirza Hasanuzzaman, Md. Rakib Hossain Raihan, Farzana Nowroz, Masayuki Fujita

**Affiliations:** 1 Department of Agronomy, Faculty of Agriculture, Sher-e-Bangla Agricultural University, Dhaka 1207, Bangladesh; 2 Laboratory of Plant Stress Responses, Faculty of Agriculture, Kagawa University, Miki-cho, Kita-gun, Takamatsu 761-0795, Japan

**Keywords:** antioxidant defense, ionic toxicity, osmotic stress, oxidative damages, plant growth promoting rhizobacteria, salinity

## Abstract

Considering the growth-promoting potential and other regulatory roles of bacteria, we investigated the possible mechanism of the role of *Bacillus subtilis* in conferring salt tolerance in soybean. Soybean (*Glycine max* cv. BARI Soybean-5) seeds were inoculated with *B. subtilis*, either through a presoaking with seeds or a direct application with pot soil. After 20 days of sowing, both the seed- and soil-inoculated plants were exposed to 50, 100, and 150 mM of NaCl for 30 days. A clear sign of oxidative stress was evident through a remarkable increase in lipid peroxidation, hydrogen peroxide, methylglyoxal, and electrolyte leakage in the salt treated plants. Moreover, the efficiency of the ascorbate (AsA)–glutathione (GSH) pathways was declined. Consequently, the plant growth, biomass accumulation, water relations, and content of the photosynthetic pigments were decreased. Salt stress also caused an increased Na^+^/K^+^ ratio and decreased Ca^2+^. On the contrary, the *B. subtilis* inoculated plants showed increased levels of AsA and GSH, their redox balance, and the activities of the AsA–GSH pathway enzymes, superoxide dismutase, catalase, glutathione peroxidase, glutathione *S*-transferase, and peroxidase. The *B. subtilis* inoculated plants also enhanced the activities of glyoxalase enzymes, which mitigated methylglyoxal toxicity in coordination with ROS homeostasis. Besides this, the accumulation of K^+^ and Ca^2+^ was increased to maintain the ion homeostasis in the *B. subtilis* inoculated plants under salinity. Furthermore, the plant water status was uplifted in the salt treated soybean plants with *B. subtilis* inoculation. This investigation reveals the potential of *B. subtilis* in mitigating salt-induced oxidative stress in soybean plants through modulating the antioxidant defense and glyoxalase systems along with maintaining ion homeostasis and osmotic adjustments. In addition, it was evident that the soil inoculation performed better than the seed inoculation in mitigating salt-induced oxidative damages in soybean.

## 1. Introduction

Climate change exerts enormous pressure on nature, causing different types of abiotic stress, among which salinity is vital. Salt stress hinders seed germination, plant growth characteristics, photosynthesis, the plant water relationship, cell membrane integrity, and ultimately, yield contributing features [1,2]. An excessive accumulation of sodium (Na^+^) and chloride (Cl^−^) ions during salt stress causes ionic toxicity and leads to the generation of reactive oxygen species (ROS) in the plant cell [3]. These ROS include superoxide (O_2_^•–^), hydrogen peroxide (H_2_O_2_), hydroxyl radical (•OH), singlet oxygen (^1^O_2_), etc., which in excess amounts leads to oxidative stress. Moreover, oxidative stress is considered one of the most deleterious consequences of salt stress because it gives rise to the disintegration and dysfunction of biological macromolecules, which results in a disturbance in cell biology [4].

Fortunately, the plant develops defense mechanisms to check the ROS-induced damages by involving different components of the antioxidant defense system, glyoxalase system, and secondary metabolites [5]. An important defensive mechanism of the plant involves non-enzymatic components (e.g., ascorbate, AsA; glutathione, GSH; carotenoids, phenolic compounds, etc.), together with enzymes such as dehydroascorbate reductase, DHAR; monodehydroascorbate reductase, MDHAR; ascorbate peroxidase, APX; glutathione reductase, GR; catalase, CAT; superoxide dismutase, SOD; glutathione peroxidase, GPX; glutathione *S*-transferase, GST, to scavenge the ROS overproduction [6]. Methylglyoxal (MG) at a minimum concentration can act as a signaling molecule to maintain the cellular redox status, but a remarkable increment of MG content during abiotic stress results in cytotoxicity [7]; however, the enzymes of the glyoxalase system help in MG detoxification to improve a plant’s salt tolerance [8]. Therefore, it is essential to check the ROS overproduction by regulating the antioxidant defense and glyoxalase systems to cope with the salt-induced condition. 

Introducing eco-friendly techniques to attenuate salt-induced damage in a plant can minimize these detrimental effects of salt stress in a sustainable way. Microorganisms are considered biodiversity reservoirs, and favor plant growth and induce the resistance to stressors (biotic and abiotic) [9]. These are abundant in nature and can take part in enhancing the plant nutrient contents and phytoremediation along with the yield in an eco-friendly way, replacing conventional agricultural practices; therefore, this can be considered an ecological engineer in climate-smart agriculture [10]. Under adverse conditions, plant growth promoting rhizobacteria (PGPR) can build symbiotic relationships with plants to cope with existing stressors and to boost their survivability and production status. An inoculation of PGPR improves the growth attributes, photosynthesis, and plant water relation, even under excess salt exposure [11,12], and reduces the growth retardant hormone (abscisic acid) content to attenuate the damages [3]. The PGPR confirms another salt tolerance potentiality by inducing the stress-related gene expressions and salt signaling SOS1-dependent pathway [13]. Plants inoculated with PGPR also exhibit better performance in ion homeostasis with the declination of Na^+^ content and increment of beneficial ions such a K^+^, Ca^2+^, and Mg^+^ [3,11]. Stressed plants with a PGPR inoculation perform better in increasing the osmoprotectants, such as proline (Pro), glycinebetaine, total soluble solid, etc. [14], and reducing the oxidative stress indicators [3,15]. Furthermore, under stress conditions, PGPR helps in the upregulation of ROS-detoxifying enzyme activities such as SOD, POD, CAT, and APX, and the non-enzyme contents such as AsA and GSH, constitutively [11,14,16]. Among the PGPRs, *Bacillus* is one of the promising genera as it helps to improve plant growth and physiology and encourages plant survivability under stress conditions. An inoculation of *Bacillus* helps to enhance the plant growth and water holding capacity and minimizes the Na^+^ toxicity, lipid peroxidation, ethylene formation, and electrical conductivity, subsequently mitigating salt-induced injuries [16,17,18]. 

Although the promising role of *B. subtilis* in improving plant characteristics has already been demonstrated in different plant studies, its role in combating salt-induced oxidative stress has rarely been studied. Furthermore, the role of *Bacillus* through seed or soil inoculation in activating the glyoxalase system to detoxify the MG content is still unknown. To achieve the sustainable development goals and future food security, multipurpose crops can be a promising option, and soybean (*Glycine max* L.) is an excellent example; however, its production is not satisfactory due to several environmental stresses, among others. Soybean is known to be moderately salt tolerant, but a higher salt concentration for a longer duration can significantly threaten its production. We hypothesized that *B. subtilis* might mitigate high salt stress in soybean, and our present study aims to explore the adaptive features of *B. subtilis* against different levels of salt exposure in soybean, and to understand how it can attenuate oxidative stress by regulating the antioxidant defense and glyoxalase systems.

## 2. Materials and Methods

### 2.1. Plant Materials, Growth Conditions, and Stress Treatments

Healthy and uniform-sized seeds of the soybean (*G. max* cv. BARI Soybean-5), collected from Bangladesh Agricultural Research Institute were used for the study. The study was carried out in a shed house where the average day and night temperature was about 33 ± 6 °C and 27 ± 4 °C, respectively, with a photosynthetic photon flux density (PPFD) about 800–2300 μmol m^−2^ s^−1^ and a relative humidity of 72 ± 5%. The seeds were placed in a 14 L plastic pot after supplementing the recommended dose of compost, urea, triple super phosphate, muriate of potash, and boron [19]. The soil inoculation of the *B. subtilis* was completed by irrigating each pot with 5 mL of bacterial solution (ca. 5 × 10^8^ CFU) followed by well-mixing. In contrast, the seeds were inoculated with the bacteria by dipping them into the solution of *B. subtilis* for 5–10 min with a slight jerking. After 20 days of sowing (DAS), sets of plants were exposed to salt stress by irrigating 50, 100, and 150 mM NaCl solutions, and only water was irrigated to the control plants. Normal or saline water was applied regularly when the soil moisture content was below 10%. Thirty days after the stress exposure, the plant samples were collected and the relevant morphological, physiological, and biochemical parameters were measured. The experiment was carried out in a completely randomized design (CRD) with three replications. 

### 2.2. Measurement of Root Length, Shoot Length, and Stem Diameter

Five plants from each treatment were randomly selected and uprooted. Then, the roots were detached from the shoots after cutting from adjacent points where the shoots connected to the soil. After that, the root length was measured from the base to the longest branch of the root using a measuring scale. Similarly, the shoot length was also estimated from the base to the highest leaf tip of the plants. Finally, the average value of the roots and shoots lengths were presented.

The same plants from each treatment were used for measuring the stem diameter. Using slide calipers, the stem diameters were taken from three different points of each plant at the base, middle, and top. Then, the average of the points of each plant of each treatment was presented.

### 2.3. Estimation of Fresh Weight and Dry Weight

Five plants from each set of treatments were randomly pulled out with the roots. Then, the adhesive dust and soil particles were removed from the roots by washing with distilled water (dH_2_O). After that, the roots and shoots were detached from each other by cutting at the junction where the shoots touched the soil and were weighed in a digital balance for the determination of fresh weight (FW). Then, the roots and shoots were air-dried followed by being oven-dried for 72 h at 80 °C in an electric oven, and again weighed to obtain the dry weight (DW). The values of the FW and DW of the roots and shoots of the five plants were averaged. 

### 2.4. Quantification of Photosynthetic Pigments

After harvesting, 0.25 g of the fresh leaf was immersed in 10 mL of 100% ethanol and placed in a water bath for extracting the pigments. Then, the colored chromophore was spectrophotometrically observed at 664, 648, and 470 nm. Finally, the pigment contents such as chlorophyll (Chl) *a*, Chl *b*, Chl (*a*+*b*), and carotenoid (Car) were quantified following the procedure of Arnon [20]. The equations are as follows:

Chl *a* = {(12.7 × A_664_) − (2.69 × A_648_)} × V × (1000 × W)

Chl *b* = {(22.9 × A_648_) − (4.68 × A_664_)} × V × (1000 × W)

Total Chl = Chl *a* + Chl *b* = {(20.2 × A_648_) − (8.02 × A_664_)} × V × (1000 × W)

Car = {(1000 × A_470_) × V/W/1000) − (1.9 × Chl *a*) − (63.14 × Chl *b*)}/214

Here, A = optical density; V = volume of 100% ethanol (mL), and W = leaf FW (g).

### 2.5. Estimation of Leaf Relative Water Content

Following the procedure of Barrs and Weatherly [21], the RWC of the leaf was estimated. First, three leaf lamina were detached from each set of treatments and weighed for the FW. Then, the laminas were immersed into dH_2_O for 24 h in a dark place covered with filter papers of two layers and weighed a second time for the turgid weight (TW), followed by the removal of excess water with blotter paper. The third DW of the leaves was taken after oven drying for 48 h at 80 °C in an electric oven. Finally, the leaf RWC was estimated following the equation:

$$\mathrm{RWC}\;\left(\%\right)=\frac{\mathrm{FW}-\mathrm{DW}}{\mathrm{TW}-\mathrm{DW}}\times 100$$

### 2.6. Determination of Proline Content

The Pro content was determined following the procedure of Bates et al. [22]. Freshly harvested leaf (0.5 g) was homogenized with 5 mL of 3% aqueous sulfosalicylic acid in an ice-cooled mortar pestle. The homogenate was centrifuged for 15 min at 11,500× *g*, and a clear supernatant was obtained. Then, a mixture containing the supernatant (1 mL), glacial acetic acid (1 mL), and acid ninhydrin dissolved in 6 M of phosphoric acid (1 mL) was incubated for 1 h in a water bath at 100 °C. After this, the incubated mixture was cooled at room temperature, and toluene (4 mL) was added to extract the free Pro from the mixture. The absorbance of the colored chromophore was taken in a spectrophotometer at 520 nm. The calculation of the amount of free Pro was performed with a standard curve prepared from a known concentration of Pro.

### 2.7. Estimation of Electrolyte Leakage

The method of Dionisio-Sese and Tobita [23] was used to estimate the EL of the leaf. A fresh leaf of 0.5 g was harvested and placed into a plastic falcon tube containing 15 mL of dH_2_O after cutting into small pieces. The leaf sample was incubated in a water bath for 1 h at 40 °C, and the first electrical conductivity (EC_1_) was taken with an electrical conductivity meter (HI-993310, Hanna, Woonsocket, RI, USA) after cooling. Then, the solution was heated again for half an hour at 121 °C in an autoclave, and the second conductivity (EC_2_) was measured after cooling at room temperature. Finally, the leaf EL was calculated with the following formula:

$$\mathrm{EL}\;\left(\%\right)=\frac{{\mathrm{EC}}_{1}}{{\mathrm{EC}}_{2}}\times 100$$

### 2.8. Assessment of Hydrogen Peroxide Content

The procedure of Yu et al. [24] was followed to assess the content of H_2_O_2_. Using a mortar pestle, the homogenization of 0.5 g of leaf was performed with 5% trichloroacetic acid (TCA). The homogenate leaf sample was centrifuged for 12 min at 11,500× *g*, and a clear aliquot was obtained. Then, the supernatant (1 mL) was incorporated with 1 mL of 10 mM potassium–phosphate (K–P) buffer (pH 7.0) and 1 mL of 1 mM potassium iodide (KI). Then, at room temperature, the mixture was incubated for 1 h and kept in a dark place, and the absorbance was taken at 390 nm in a spectrophotometer. The final calculation was performed using an extinction coefficient of 0.28 μM^−1^ cm^−1^ to assess the amount of H_2_O_2_ content.

### 2.9. Quantification of Malondialdehyde Content

According to the technique of Heath and Packer [25], the amount of lipid peroxidation as malondialdehyde (MDA) content was quantified using a thiobarbituric acid (TBA) reagent. Fresh leaf (0.5 g) was macerated after adding 5% TCA (3 mL), and the homogenate was centrifuged for 12 min at 11,500× *g*. Then, 1 mL of the clear aliquot was incorporated with 4 mL of TBA reagent, which contained 0.5% TBA and 20% TCA solution. The composite solution of the plant sample and TBA reagent was incubated for half an hour in a water bath at 95 °C. After cooling, the optical density of the chromophore was spectrophotometrically observed at 532 nm and again at 600 nm for the non-specific value. The final calculation was performed after subtracting the non-specific value and using an extinction coefficient of 155 mM^−1^ cm^−1^. 

### 2.10. Histochemical Detection of Hydrogen Peroxide and Superoxide Anion

Spots of H_2_O_2_ and O_2_^•−^ were detected following the procedure of Chen et al. [26]. For the localization of the spots of H_2_O_2_ and O_2_^•−^, 0.1% of 3,3′ diaminobenzidine (DAB) and nitro blue tetrazolium chloride (NBT) solutions were used, respectively. Leaves were plucked and immersed into the solution of DAB and NBT, maintaining a pH of 3.8. Then, the leaves were incubated until the spots were reddish-brown for DAB and dark blue for NBT. Then, the leaves were washed with boiling 100% ethanol until the leaves were discolored, and photographs were taken.

### 2.11. Estimation of Methylglyoxal Content

The method of Wild et al. [27] was followed for the estimation of the MG content. The homogenization of 0.25 g of fresh leaf was performed using 3 mL of 5% perchloric acid and centrifuged for 12 min at 11,500× *g*. The supernatant was discolored with the activated charcoal by centrifuging for 12 min at 11,500× *g*. The discolored supernatant was then neutralized with saturated sodium carbonate (Na_2_CO_3_) solution at pH 7.0. After this, 500 µL of neutralized supernatant, 10 µL of *N*-acetyl-*L*-cysteine, and 480 µL of sodium–phosphate (Na–P) buffer were incubated for 20 min for the formation of a *N*-α-acetyl-S-(1-hydroxy-2-oxoprop-1-yl) cysteine bond. Finally, the optical density of the mixture was taken in a spectrophotometer at 288 nm, and the calculation was performed by plotting the absorbance value against a standard curve of a known concentration of MG. 

### 2.12. Quantification of Na^+^, K^+^, and Ca^2+^ Content

The Na^+^, K^+^, and Ca^2+^ ion contents were quantified by using a compact Na^+^ meter (Laquatwin-Na-11, HORIBA Advanced Techno Co., Romeoville, IL, USA), compact K^+^ meter (Laquatwin-K-11, HORIBA Advanced Techno Co., Romeoville, IL, USA), and compact Ca^2+^ meter (Laquatwin-Ca-11, HORIBA Advanced Techno Co., Romeoville, IL, USA), respectively. An adequate amount of leaf from each treatment was harvested and washed with dH_2_O to remove the adhesive dust. Then, the cell sap was squeezed with the sap extractor and applied to the respective ion-meter sensor. The final reading was taken when a smiley sign was seen on the screen, and the amount of ion content was expressed as ppm.

### 2.13. Determination of Ascorbate and Glutathione Content 

Half of a gram of the leaf was used to determine the ascorbate and glutathione content. The leaves were macerated with 5% metaphosphoric acid containing 1 mM of ethylenediaminetetraacetic acid (EDTA) and centrifuged at 11,500× *g* for 15 min. Then, the clear supernatant was obtained and used to estimate the AsA and GSH content. Following the procedure of Huang et al. [28], the content of reduced and total AsA were determined. To determine the reduced AsA, the supernatant was neutralized with 0.5 M of the K–P buffer (pH 7.0) and dH_2_O, whereas 0.1 M dithiothreitol (DTT) was used for estimating the total AsA content instead of dH_2_O. Then, the optical density was assayed at 265 nm in a spectrophotometer using 100 mM of K–P buffer (pH 6.5) and 0.5 unit of ascorbate oxidase (AO). The final quantification of the content of the AsA and total AsA was performed by plotting the values against a standard curve. By subtracting the value of the reduced AsA from the total AsA, the DHA content was calculated. The contents of AsA and DHA were expressed as nmol g^−1^ FW. 

The method of Hasanuzzaman et al. [5] was followed in the determination of the GSH content. Neutralization of the supernatant was performed with 0.5 M of K–P buffer (pH 7.0) and dH_2_O for the total GSH, whereas, 2–vinylpyridine along with the K–P buffer was added for the determination of the GSSG. For observing the optical density at 412 nm in a spectrophotometer, the neutralized mixture was oxidized with 5,5-dithio-bis(2-nitrobenzoic acid) (DTNB) followed by a reduction with nicotinamide adenine dinucleotide phosphate (NADPH) and GR. The absorbance was plotted against a standard curve to estimate the GSSG and total GSH, and the measurement of the GSH content was performed by deducting the value of the GSSG from the total GSH. The contents of the GSH and GSSG were expressed as nmol g^−1^ FW.

### 2.14. Extraction of Enzyme and Estimation of Free Protein

For extracting the enzymes, the protocol of Hasanuzzaman et al. [5] was used. An extraction buffer prepared with L-ascorbic acid (Asc; 1 mM), KCl (100 mM), 0.5 M of K–P buffer (pH 7.0), β-mercaptoethanol (5 mM), and glycerol (10%), and 1 mL of buffer was used for the homogenization of 0.5 g of leaf in an ice-cooled mortar pestle. Then, the homogenate was transferred to an Eppendorf tube and centrifuged for 12 min at 11,500× *g* at 4 °C. Then, a clear aliquot was obtained and preserved for the further estimation of the free protein content and antioxidant enzyme activity.

The concentration of protein was measured followed by the method of Bradford [29]. The Bradford reagent was made using Coomassie brilliant blue (CBB G-250), 100% ethanol, 85% ortho-phosphoric acid, and dH_2_O with continuous agitation, and the mixture was also filtered. Then, the extracted supernatant of 5 µL was mixed with the 5 mL of Bradford reagent, and the optical density was taken at 595 nm. The final estimation of the protein concentration was performed by plotting the values against a standard curve prepared from bovine serum albumin.

### 2.15. Determination of Enzymatic Activities

The procedure of El-Shabrawi et al. [30], which is based on the xanthine-xanthine oxidase system was used for the assessment of the superoxide dismutase (SOD; EC: 1.15.1.1) activity. The reaction buffer of K–P buffer (50 mM, pH 7.0), NBT (2.24 mM), xanthine (2.36 mM), and xanthine oxidase (0.1 unit), were needed for the observation of the SOD activity. A continuous agitation of xanthine was required for the activation of the reaction, and the activity was observed at 560 nm spectrophotometrically. Finally, the SOD activity was computed and expressed as U mg^−1^ protein.

The catalase (CAT; EC: 1.11.1.6) activity was determined following the technique of Hasanuzzaman et al. [5]. A reaction buffer of H_2_O_2_ (15 mM) and K–P buffer (50 mM, pH 7.0) were needed for assaying the activity. The enzyme was degraded upon the inclusion of H_2_O_2,_ and the absorbance was taken at 240 nm in a spectrophotometer. An extinction coefficient of 39.4 M^−1^ cm^−1^ was used for the calculation of the CAT activity and expressed as µmol min^−1^ mg^−1^ protein.

The method of Nakano and Asada [31] was used for the assay of the ascorbate peroxidase (APX; EC: 1.11.1.11) activity. A reaction buffer consisting of Asc (0.5 mM), EDTA (0.1 mM), and K–P buffer (15 mM, pH 7.0) was used to assess the enzyme activity where H_2_O_2_ (0.1 mM) was added at last to initiate the reaction. The activity was observed at 290 nm spectrophotometrically, and an extinction coefficient (2.8 mM^−1^ cm^−1^) was used for the calculation. The unit of APX activity was expressed as µmol min^−1^ mg^−1^ protein.

The technique of Hasanuzzaman et al. [5] was followed for the determination of the glutathione reductase (GR; EC: 1.6.4.2) activity. The assay buffer consisted of K–P buffer (0.1 M, pH 7.8) and NADPH (0.2 mM), and EDTA (1 mM) was required for observing the activity where GSSG (1 mM) was added to the enzymes as a precursor of the reaction. The activity was observed at 340 nm spectrophotometrically. The GR activity was computed with an extinction coefficient of 6.2 mM^−1^ cm^−1^ and expressed as µmol min^−1^ mg^−1^ protein.

The activity of the monodehydroascorbate reductase (MDHAR; EC: 1.6.5.4) was observed according to the procedure of Hossain et al. [32]. An assay buffer containing Tris-HCl (50 mM, pH 7.5), Asc (2.5 mM), and NADPH (0.2 mM) was used for the observation of the MDHAR activity where AO (1 unit) was included for the activation of the reaction. The activity was observed in a spectrophotometer at 340 nm. An extinction coefficient of 6.2 mM^−1^ cm^−1^ was used for the calculation of the activity, and it was expressed as nmol min^−1^ mg^−1^ protein.

The dehydroascorbate reductase (DHAR; EC: 1.8.5.1) activity was assayed following the method of Nakano and Asada [31]. A reaction buffer was prepared with GSH (2.5 mM), EDTA (0.1 mM), and K–P buffer (50 mM, pH 7.0), where the addition of DHA (0.1 mM) was needed for the initiation of the reaction. The DHAR activity was assessed spectrophotometrically at 265 nm, and an extinction coefficient of 14 mM^−1^ cm^−1^ was used to estimate the activity. The DHAR activity was expressed as nmol min^−1^ mg^−1^ protein.

The assessment of peroxidase (POD; EC: 1.11.1.7) activity was completed with the Hemeda and Klein [33] technique. The activity was observed with the reaction buffer of guaiacol (1.5 mM) and K–P buffer (0.5 M, pH 7.0). Here, guaiacol acted in the role of electron donor for the oxidation and the reaction was initiated with the inclusion of H_2_O_2_ (30 mM). Then, the activity was observed spectrophotometrically at 470 nm, and the estimation of the activity was performed with an extinction coefficient of 26.6 mM^−1^ cm^−1^. The POD activity was expressed as µmol min^−1^ mg^−1^ protein.

The glutathione peroxidase (GPX; 1.11.1.9) activity was assayed with the procedure of Hasanuzzaman et al. [5] with slight modifications from Elia et al. [34]. An assay buffer consisting of NADPH (0.12 mM), GSH (2 mM), GR (1 unit), sodium azide (NaN_3_; 1 mM), K–P buffer (100 mM, pH 7.0), and EDTA (1 mM) was needed to observe the activity. The inclusion of H_2_O_2_ (10.50 mM) was required for the initiation of the reaction. The enzymatic oxidation of the NADPH was observed spectrophotometrically at 340 nm. The GPX activity was quantified with an extinction coefficient of 6.62 mM^−1^ cm^−1^ and expressed as nmol min^−1^ mg^−1^ protein.

The procedure of Hasanuzzaman et al. [5] was followed for the estimation of the glutathione *S*-transferase (GST; EC: 2.5.1.18) activity. An assay buffer of K–P buffer (0.25 M, pH 6.5), GSH (1.5 mM), and 1-chloro-2,4-dinitrobenzene (CDNB; 1 mM) was used to observe the activity at 340 nm with a spectrophotometer. The breakdown of the enzymes initiated with the inclusion of CDNB. The activity was calculated with the extinction coefficient of 9.6 mM^−1^ cm^−1^ and expressed as nmol min^−1^ mg^−1^ protein.

The glyoxalase I (Gly I; EC: 4.4.1.5) activity was assessed following the technique of Hasanuzzaman et al. [5]. A reaction buffer of Na–P buffer (100 mM), GSH (100 mM), and magnesium phosphate (MgSO_4_; 16 mM) was used to evaluate the activity at 240 mM spectrophotometrically, where the reaction was commenced with the addition of MG (35 mM). The calculation of the Gly I activity was performed with 3.37 mM^−1^ cm^−1^ as an extinction coefficient and expressed as µmol min^−1^ mg^−1^ protein.

Following the method of Principato et al. [35], the activity of glyoxalase-II (Gly-II; EC: 3.1.2.6) was quantified. An assay buffer containing Tris-HCl buffer (100 mM, pH 7.2), DTNB (0.2 mM), and *S*-_D_-lactoylglutathione (SLG; 1 mM) was used to observe the activity at 412 nm in a spectrophotometer. The enzymatic reaction commenced after the addition of SLG, and the estimation was performed with an extinction coefficient of 13.6 mM^−1^ cm^−1^. The activity was expressed as µmol min^−1^ mg^−1^ protein.

### 2.16. Statistical Analysis

The statistical analysis of the experimental data was completed with the CoStat v.6.400 computer-based software [36]. The mean ± SD values were computed from three replications, and the treatments were compared after applying a Tukey’s HSD test at a 5% level of significance (*p* ≤ 0.05).

## 3. Results

### 3.1. Plant Growth and Biomass

A dose-dependent reduction in the root length was observed due to NaCl (Table 1). Upon exposure to 50, 100, and 150 mM of NaCl, the root length was decreased by 18, 25, and 31%, respectively, compared to the controls (Table 1); however, the shoot length was reduced only under 100 and 150 mM NaCl, which was 24 and 30%, respectively, compared to the controls (Table 1). Soil inoculation with *B. subtilis* improved the root length in all cases (Table 1). In the case of the shoot length, the seed and soil inoculation uplifted the length only at 100 mM NaCl by 24 and 22%, respectively, compared to the corresponding NaCl treated only (Table 1). A remarkable decrease in the stem diameter was recorded at 100 and 150 mM NaCl, which was 17 and 34%, respectively, compared to the controls (Table 1). Conversely, the diameter was improved only at 150 mM NaCl which was 30 and 25% due to the seed and soil inoculation of *B. subtilis*, respectively, compared to the NaCl alone (Table 1).

The plant biomass was decreased in all salt levels (Table 1). The highest reduction of the root FW (59%) and shoot FW (73%) was recorded at 150 mM NaCl. (Table 1). Both the seed and soil inoculation with *B. subtilis* improved the soybean root and shoot FW, in all cases (Table 1). At 50, 100, and 150 mM NaCl, the reduction of the root DW was 45, 58, and 60%, respectively, and the shoot DW was 48, 54, and 73%, respectively, compared to the control plants (Table 1). The root DW was uplifted by the *B. subtilis* seed inoculation only under 100 and 150 mM NaCl by 41 and 26%, respectively (Table 1). In contrast, the soil inoculated with *B. subtilis* recovered the DW of the root in all cases (Table 1). Upon 50 and 150 mM NaCl, the seed inoculation increased the shoot DW by 26 and 50%, respectively, compared to the corresponding salt stress alone (Table 1).

### 3.2. Photosynthetic Pigments

**Figure 1:**
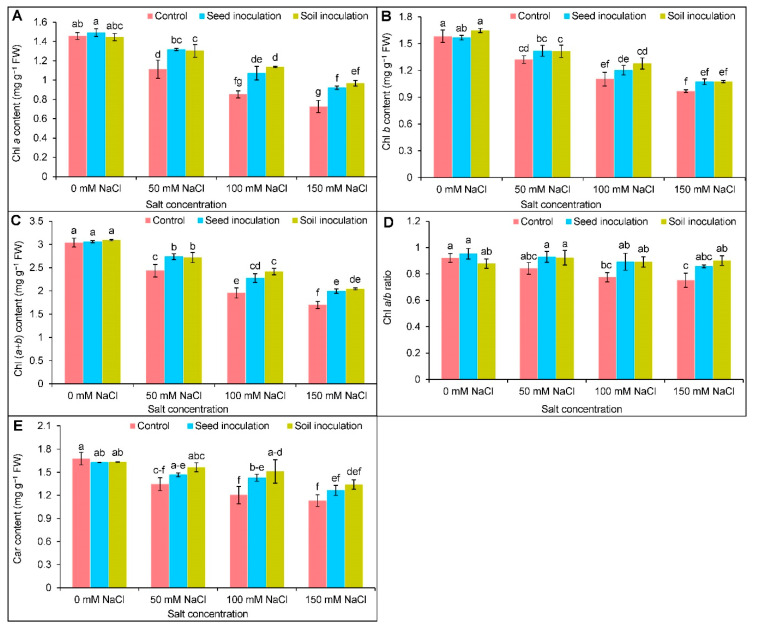
Effect of *Bacillus subtilis* in response to different concentrations of salt stress on the contents of Chl *a* (**A**), Chl *b* (**B**), Chl (*a+b*) (**C**), Chl *a/b* (**D**), and Car (**E**) of soybean (*Glycine max* cv. BARI Soybean-5). Values in a column are mean ± SD. Different letters on each bar indicate significant differences among the treatment after applying a Tukey’s HSD test at *p* ≤ 0.05.

Compared to the controls, a significant reduction was observed in the Chl *a*, Chl *b*, Chl (*a*+*b*), and Car contents of the salt-induced plants (Figure 1A–C,E). Upon NaCl exposure at 50, 100, and 150 mM levels, the reduction of the Chl *a* content was 24, 42, and 50%, respectively (Figure 1A), whereas the Chl *b* content was declined by 17, 31, and 39%, respectively, compared to the controls (Figure 1A, B). Likewise, the Chl (*a*+*b*) content was also reduced in a dose-dependent manner (Figure 1C), whereas the ratio of Chl *a/b* was only decreased by 16 and 19%, at 100 and 150 mM NaCl, respectively, in comparison to the controls (Figure 1D). The Car content was decreased by 20, 29, and 33%, respectively (Figure 1E); however, in increasing the Chl *a* and Chl (*a*+*b*) contents, the inoculation with *B. subtilis* in both forms resulted in a better performance under salt exposure (Figure 1A,C). Under NaCl exposure, the Chl *b* content was increased by the soil inoculation only at 100 mM NaCl by 17% (Figure 1B). Only upon 150 mM of NaCl exposure, the *B. subtilis* soil inoculation boosted the Chl *a/b* ratio by 20% compared to the respective stressed plant alone (Figure 1D). Both the seed and soil inoculation increased the Car content only under 100 mM of NaCl by 20 and 22%, respectively, compared to being NaCl stressed only (Figure 1E).

### 3.3. Ion Contents

**Figure 2:**
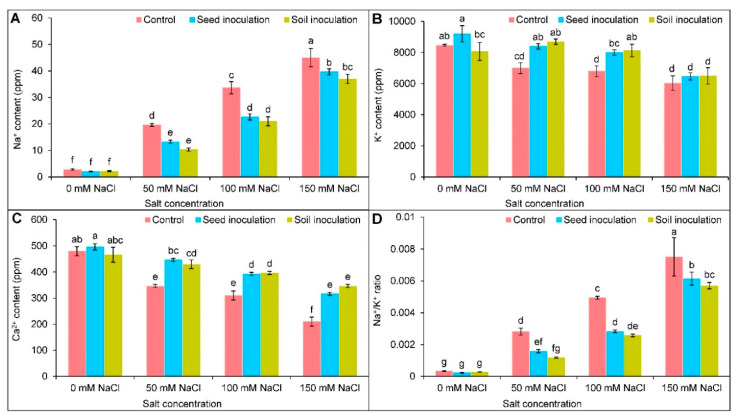
Effect of *Bacillus subtilis* in response to different concentrations of salt stress on the contents of Na^+^ (**A**), K^+^ (**B**), Ca^2+^ (**C**), and Na^+^/K^+^ ratio (**D**) of soybean (*Glycine max* cv. BARI Soybean-5). Values in a column are mean ± SD. Different letters on each bar indicate significant differences among the treatment after applying a Tukey’s HSD test at *p* ≤ 0.05.

With the increasing salt concentrations, increased Na^+^ content and decreased K^+^ content were observed (Figure 2A,B); however, under 50, 100, and 150 NaCl, the seed inoculation with *B. subtilis* downregulated the Na^+^ content by 32, 33, and 12%, respectively, whereas the soil inoculation reduced it by 47, 38, and 18%, respectively, in comparison to the respective NaCl stressed plants only (Figure 2A). A further recovery of the K^+^ content was observed at 50 and 100 mM NaCl with the seed inoculation of *B. subtilis* by 20 and 18%, respectively, whereas the enhancement was 24 and 20%, respectively, with the *B. subtilis* soil inoculation compared to the corresponding salt stressed plants (Figure 2B). The Na^+^/K^+^ ratio was also increased in a dose-dependent manner upon salt exposure (Figure 2D). On the contrary, under all NaCl treatments, the seed and soil inoculation successfully decreased the ratio (Figure 2D). The Ca^2+^ content was reduced at 50, 100, and 150 mM of NaCl by 28, 35, and 56%, respectively, compared to the controls (Figure 2C). A further improvement of the Ca^2+^ content, under 50, 100, and 150 mM of NaCl was observed by both the seed and soil inoculations compared to the salt stressed plant alone (Figure 2C).

### 3.4. Physiological Attributes

#### 3.4.1. Relative Water Content

**Figure 3:**
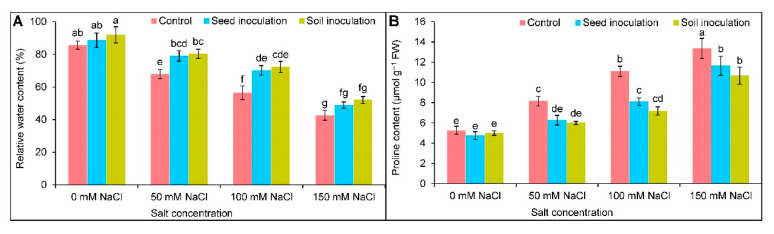
Effect of *Bacillus subtilis* in response to different concentrations of salt stress, on the relative water content (**A**) and proline content (**B**) of soybean (*Glycine max* cv. BARI Soybean-5). Values in a column are mean ± SD. Different letters on each bar indicate significant differences among the treatment after applying a Tukey’s HSD test at *p* ≤ 0.05.

The decreasing trend of RWC upon exposure stress was observed in a dose-dependent manner. It was reduced by 18, 29, and 43% under 50, 100, and 150 mM of NaCl, respectively, in comparison to the controls (Figure 3A); however, under 50 and 100 mM NaCl, the seed inoculation with *B. subtilis* recovered the RWC by 11 and 14%, respectively. In contrast, the soil inoculation improved it by 12 and 16%, respectively, compared to the corresponding NaCl affected plants only (Figure 3A).

#### 3.4.2. Proline Content

As expected, the Pro content was uplifted due to salt exposures in comparison to the control (Figure 3B). At 50, 100, and 150 mM NaCl stress, the seed inoculation resulted in a reduced Pro content by 23, 27, and 13%, respectively, and the soil inoculation reduced the content by 27, 36, and 20%, respectively, compared to the respective NaCl stressed alone (Figure 3B).

### 3.5. Oxidative Stress Indicators

**Figure 4:**
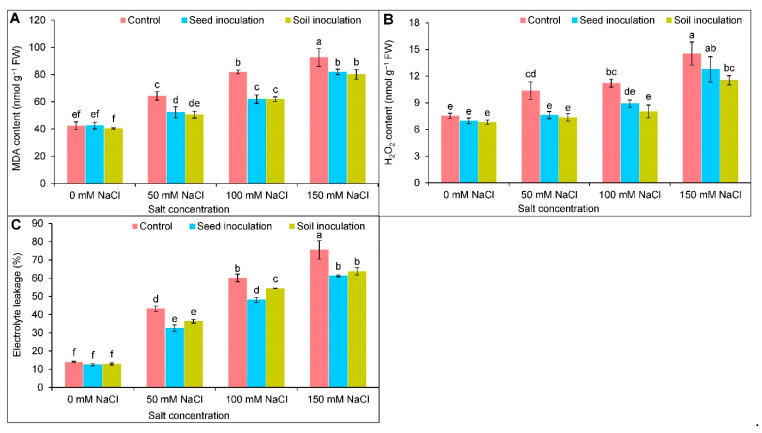
Effect of *Bacillus subtilis* in response to different concentrations of salt stress on the contents of MDA (**A**), H_2_O_2_ (**B**), and electrolyte leakage (**C**) of soybean (*Glycine max* cv. BARI Soybean-5). Values in a column are mean ± SD. Different letters on each bar indicate significant differences among the treatment after applying a Tukey’s HSD test at *p* ≤ 0.05.

As a result of salt accumulation, an increasing trend was observed in the MDA and H_2_O_2_ contents (Figure 4A,B). Under 50, 100, and 150 mM of NaCl, the MDA content was markedly uplifted by 52, 94, and 119%, respectively (Figure 4A), and the H_2_O_2_ content by 37, 49, and 93%, respectively, compared to the controls (Figure 4B). The MDA content was decreased at 50, 100, and 150 mM of NaCl through the *B. subtilis* seed inoculation by 19, 25, and 12%, and the soil inoculation by 22, 25, and 13%, respectively, compared to the stressed plants alone (Figure 4A). At 50 and 100 mM of NaCl, the seed inoculation of *B. subtilis* decreased the H_2_O_2_ content by 27 and 21%, respectively, compared to the NaCl treated alone (Figure 4B). In contrast, the soil inoculation of *B. subtilis* under 50, 100, and 150 mM of NaCl, reduced the H_2_O_2_ content by 29, 29, and 21%, respectively (Figure 4B). Salt stress increased the EL in a dose-dependent manner, and compared to the controls, at 50, 100, and 150 mM of NaCl, the EL was increased by 3.1-fold, 4.32-fold, and 5.42-fold, respectively (Figure 4C). Conversely, a decreasing trend of the EL was observed in the seed and soil inoculations with *B. subtilis* under salt stress (Figure 4C).

**Figure 5:**
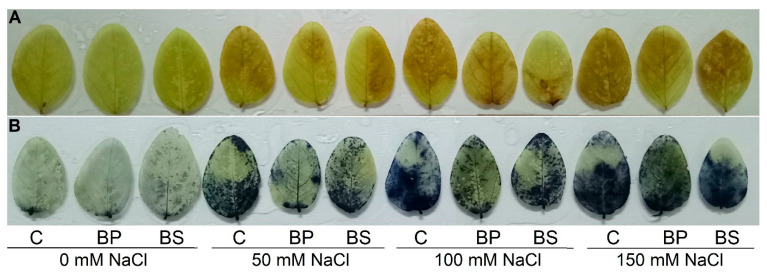
Localization of the spots of H_2_O_2_ (**A**) and O_2_^•−^ radicals (**B**) on the leaves of soybean (*Glycine max* cv. BARI Soybean-5) as affected by *Bacillus subtilis* in response to different concentrations of salt stress. Here, C, BP, and BS indicate the control, seed inoculation with *Bacillus* *subtilis*, and soil inoculation with *Bacillus subtilis*, respectively.

To observe the generation of ROS (H_2_O_2_ and O_2_^•−^), spot localization was performed by staining. Remarkable changes were detected in all the salt treatments (Figure 5A,B). With the increase in salt concentrations, the brown area of H_2_O_2_ and dark blue regions of O_2_^•−^ became more prominent compared to the control (Figure 5A,B). Conversely, the inoculation of *B. subtilis* considerably took part in protecting the leaves from an overproduction of ROS, which was evident by reduced spots compared to the NaCl-affected alone (Figure 5A,B).

### 3.6. Ascorbate-Glutathione Pool

**Figure 6:**
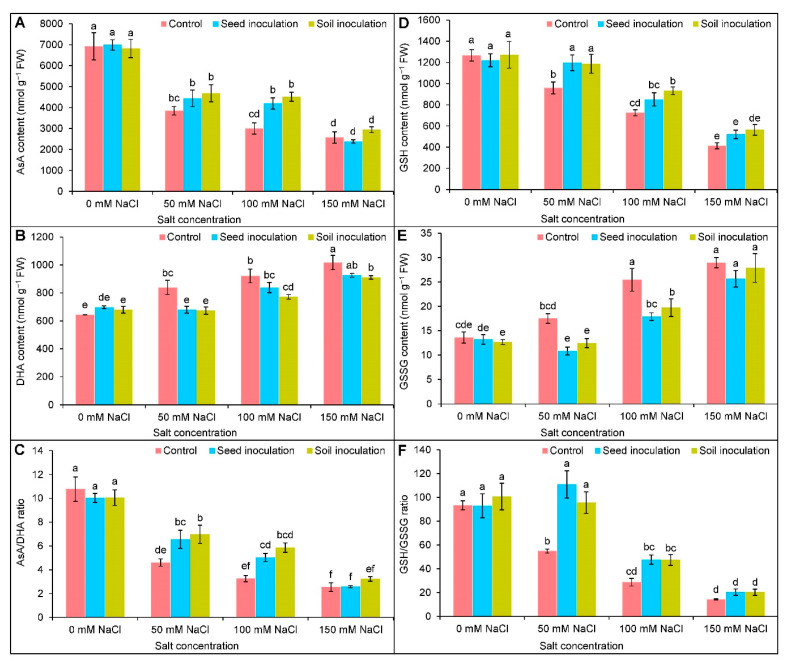
Effect of *Bacillus subtilis* in response to different concentrations of salt stress on the contents of AsA (**A**), DHA (**B**), AsA/DHA ratio (**C**), GSH (**D**), GSSG (**E**), and GSH/GSSG (**F**) of soybean (*Glycine max* cv. BARI Soybean-5). Values in a column are mean ± SD. Different letters on each bar indicate significant differences among the treatment after applying a Tukey’s HSD test at *p* ≤ 0.05.

With the increasing salt concentrations, the rate of AsA and GSH contents was reduced (Figure 6A,D). In contrast, the DHA content was increased compared to the control plants (Figure 6B). The GSSG content was uplifted by 87 and 113% under 100 and 150 mM of NaCl, respectively, in comparison to the controls (Figure 6E); therefore, both the ratios of AsA/DHA and GSH/GSSG were decreased under different levels of salt concentrations (Figure 6C,F). The decreased AsA content was uplifted only under 100 mM of NaCl by 40 and 51% due to seed and soil inoculation, respectively (Figure 6A). The DHA content was decreased (19%) by the seed inoculation only at 50 mM of NaCl in comparison to the NaCl-stressed alone example. In contrast, the soil inoculation performed better in reducing the DHA content in all cases (Figure 6B). The ratio of AsA/DHA was uplifted only under 50 and 100 mM of NaCl by the seed and soil inoculations of *B. subtilis* compared to the respective NaCl-stressed only (Figure 6C).

The decreased GSH content was increased by seed inoculation only at 50 mM of NaCl by 25%. Similarly, the soil inoculation improved the GSH content by 24 and 29% under 50 and 100 mM of NaCl, respectively (Figure 6D). Under 50 and 100 mM of NaCl, the seed and soil inoculation with *B. subtilis* resulted in a decreased GSSG content (Figure 6E). At 50 mM of NaCl, the ratio of GSH/GSSG was increased by 102 and 74% for the seed and soil inoculations, respectively, compared to the corresponding NaCl stressed only (Figure 6F).

### 3.7. Antioxidant Enzymes Activities

**Figure 7:**
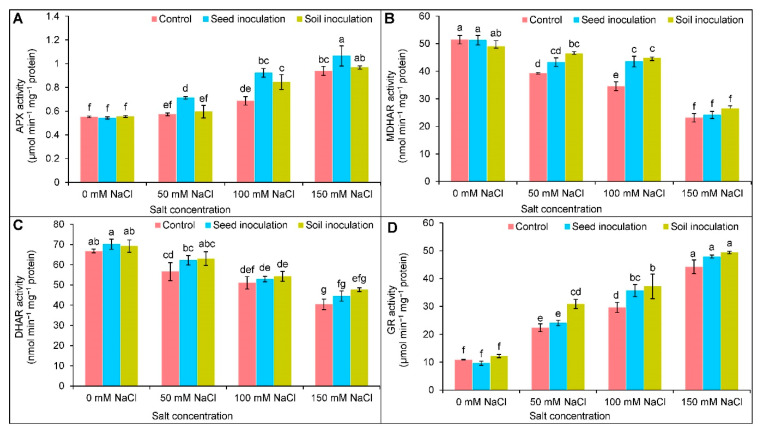
Effect of *Bacillus subtilis* in response to different concentrations of salt stress on the activities of APX (**A**), MDHAR (**B**), DHAR (**C**), and GR (**D**) of soybean (*Glycine max* cv. BARI Soybean-5). Values in a column are mean ± SD. Different letters on each bar indicate significant differences among the treatment after applying a Tukey’s HSD test at *p* ≤ 0.05.

Compared to the controls, the activity of APX was increased by 25 and 71% under 100 and 150 mM of NaCl, respectively (Figure 7A). Under 50, 100, and 150 mM of NaCl, the *B. subtilis* seed inoculation increased the activity of the APX further by 25, 33, and 14%, respectively, in comparison to the corresponding salt-stressed only examples (Figure 7A). The soil inoculation improved the APX activity only at 100 mM of NaCl by 23% (Figure 7A). Under salt exposure, the MDHAR and DHAR activities were decreased with increasing salt levels (Figure 7B,C). The MDHAR activity was improved through seed inoculation only at 100 mM of NaCl by 26% and soil inoculation at 50 and 100 mM of NaCl by 18 and 30%, respectively (Figure 7B). There were no significant changes in the DHAR activity exhibited by inoculations under NaCl (Figure 7C). The GR activity was increased in a dose-dependent manner when imposed to salt stress (Figure 7D). The activity was further uplifted by seed inoculation only at 100 mM of NaCl and by soil inoculation at 50 and 100 mM of NaCl compared to the respective NaCl treated only (Figure 7D).

**Figure 8:**
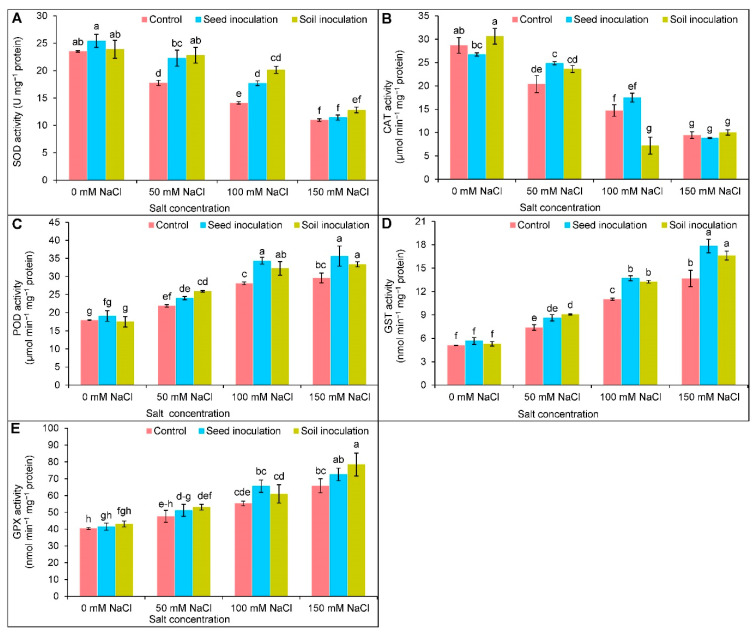
Effect of *Bacillus subtilis* in response to different concentrations of salt stress on the activities of SOD (**A**), CAT (**B**), POD (**C**), GST (**D**), and GPX (**E**) of soybean (*Glycine max* cv. BARI Soybean-5). Values in a column are mean ± SD. Different letters on each bar indicate significant differences among the treatment after applying a Tukey’s HSD test at *p* ≤ 0.05.

Both the activities of SOD and CAT were reduced in a dose-dependent manner at different levels of salt (Figure 8A,B). At 50 and 100 mM of NaCl, the seed and soil inoculated *B. subtilis* increased the activity of SOD compared to the corresponding NaCl-stressed alone (Figure 8A). The CAT activity was increased (22%) by the seed inoculation only at 50 mM of NaCl whereas, the soil inoculation decreased the activity (51%) only under 100 mM of NaCl compared to the corresponding NaCl treated alone (Figure 8B). The activities of POD and GST were increased with increasing salt concentrations in comparison to the control plants (Figure 8C,D). The activities were further increased through seed inoculation at 100 and 150 mM salt levels and the soil inoculation in all cases compared to the salt stressed alone (Figure 8C,D). The GPX activity was uplifted by 37 and 63% under 100 and 150 mM of NaCl, respectively, compared to the controls (Figure 8E). Further improvement of the GPX activity was found by soil inoculation at 150 mM of NaCl by 19% in comparison to the NaCl stressed alone (Figure 8E).

### 3.8. Glyoxalase System

**Figure 9:**
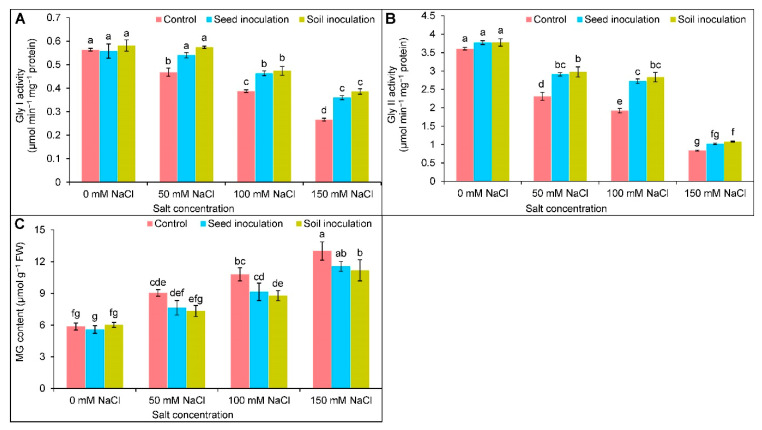
Effect of *Bacillus subtilis* in response to different concentrations of salt stress on the activities of Gly I (**A**), Gly II (**B**), and MG content (**C**) of soybean (*Glycine max* cv. BARI Soybean-5). Values in a column are mean ± SD. Different letters on each bar indicate significant differences among the treatment after applying a Tukey’s HSD test at *p* ≤ 0.05.

A substantial reduction in Gly I and Gly II activities was exhibited due to salt exposure (Figure 9A,B). At 50, 100, and 150 mM of NaCl, the Gly I activity was reduced by 16, 30, and 52%, respectively; similarly, the Gly II activity was declined by 36, 47, and 77%, respectively, compared to the controls (Figure 9A,B). Under 50, 100, and 150 mM of NaCl, the *B. subtilis* seed and soil inoculations further increased the Gly I activity (Figure 9A). Whereas the Gly II activity was upgraded by 26 and 42% only at 50 and 100 mM of NaCl, respectively, as a result of the seed inoculation (Figure 9B). The soil inoculation uplifted the Gly II activity in the NaCl exposures, compared to the respective salt-stressed alone (Figure 9B).

An increasing trend was observed in the MG content upon salt exposure (Figure 9C). Compared to the controls, the MG content was enhanced by 54, 84, and 122% under 50, 100, and 150 mM of NaCl, respectively (Figure 9C); although, the MG content was statistically similar in the case of the seed inoculation and controls, where in contrast, the soil inoculation of *B. subtilis* decreased the content by 19 and 14% only under 100 and 150 mM of NaCl, respectively, in comparison to the respective NaCl treated plants alone (Figure 9C).

## 4. Discussion

The present study exhibited plant growth inhibition as evidenced by the reduced length of the roots and shoots, stem diameter, and biomass acquisition in salt affected soybean plants. The cell differentiation and elongation were adversely affected when exposed to salt stress that restricted the growth of the plants [37,38]; however, recent advances in microbial research have identified some PGPR-inducing plant salt tolerance [12,39]. These PGPR upregulate phytohormone production, such as indole acetic acid (IAA), which is one of the vital growth accelerating hormones under stress [40]. The inoculation of *Pseudomonas, Bacillus*, and *Brevibacterium* elevated the production of IAA and promoted the plant growth and tolerance to salt stressed plants [12,41,42]. Moreover, microbes are potent for solubilizing unavailable minerals into available forms that can readily be taken up by plants and augment plant growth [43]. Shultana et al. [14] evaluated the performance of *Bacillus* strains on the phosphorus (P) and potassium (K) solubilizing capacity and found a stimulated availability of P and K under saline conditions. Moreover, siderophore production by *Bacillus* converts unavailable forms of iron (Fe) into available forms and improves the root length, plant height, and stem diameter of the *Capsicum annuum* under salt stress [44].

Under salinity, the augmented activity of the chlorophyllase enzymes destabilizes photosynthetic pigment proteins. Excessive amounts of Na^+^ ions have also altered the ultrastructure of chloroplast and, consequently, hampered Chl biosynthesis and photoassimilates production [45,46]. In our experiment, photosynthetic pigments such as Chl *a*, Chl *b*, and Car accumulation were restored in the plants with the inoculation of *B. subtilis* under salt stress. The restoration of the photosynthetic pigments in the inoculated plants might have been due to the mineral solubilization of Fe and magnesium (Mg) by the microbes under stress [47,48]. Magnesium occupies the central tetrapyrrole ring of Chl *a* and Chl *b*, which are the vital light-harvesting apparatus; thus, a decrease in Mg adversely affects the photosynthetic rate [49]. At the same time, siderophores augment the Fe availability, which influences the synthesis of Chl and transfers electrons to the photosynthetic and respiratory processes of plants [50,51,52].

The immediate effect of soil salinization is the Na^+^-induced K^+^ efflux from plants. When exposed to salt stress, higher amounts of Na^+^ deposition lead to lower contents of K^+^ and Ca^2+^ in soybean; however, the *B. subtilis*-inoculated plants showed improved K^+^ and Ca^2+^ ions accumulation in the salt-induced soybean. The possible mechanism of PGPR in alleviating the toxic effects of Na^+^ is the secretion of exopolysaccharides (EPS), which helps to stabilize plant-microbe interactions and induce systemic resistance to environmental stresses [53]. The EPS-producing microbes restrict the Na^+^ deposition under salinity and elucidate the NaCl-induced damages [11,54]. Moreover, an inoculation with *Bacillus* sp. in the *G. max* [52], *C. annuum* [44], and *Triticum aestivum* [12] mitigated an accumulation of Na^+^, thereby maintaining the ionic homeostasis of the plants.

Under salinity, the reduction in the K^+^ content diminished the stomatal opening and closing, as this is a vital element in adjusting the osmotic balance [55]. Moreover, a hindrance in the water uptake due to a lower water potential accelerates apoptosis under saline conditions. To tackle this situation, a plant accumulates more compatible osmolyte (Pro) to mitigate the osmotic shock to the plant [38]. In the current study, the soybean plants exposed to salt stress showed a reduced RWC and accelerated Pro content, indicating salt-induced osmotic stress; however, the *B. subtilis* inoculation, interestingly, upgraded the RWC of the salt treated soybean plants and declined the Pro accumulation. This might have been due to the production of EPS by PGPR, which generates a protective biofilm within the cells of microbes. These biofilms can regulate nutrient and water consumption from the rhizosphere, performing as a strong osmo-regulating tool under stress [56]. Ji et al. [18] demonstrated that *Bacillus* inoculation in salt affected *T. aestivum* seedlings mitigated cellular dehydration through regulating the osmolytes accumulation, such as Pro and soluble sugars. Similarly, by inoculating *Bacillus* in the salt stressed *T. aestivum* [57], the salt-induced osmotic stress was mitigated by lowering the Pro synthesis, which is in congruence with the present findings.

The overproduced ROS has a pernicious effect on the cellular organelles that destroy lipids, proteins, and carbohydrates; however, ROSs are not harmful to a certain level and act as a signaling cascade [4]. A notable increase in H_2_O_2_ disintegrates the membrane structure by increasing the lipid peroxidation (marked by the higher MDA content) in salt affected soybean plants. Moreover, the augmented production of H_2_O_2_ and MDA contents led to an increase in the EL in the plants due to salt exposure in the current study; however, the PGPR has a protective role in ameliorating ionic and osmotic stress by producing EPS that restricts the direct contact and translocation of Na^+^ in plants. The EPS also acts as a biofilm to adjust the osmotic imbalance; thus, enhancing the cellular integrity [14,57]. The maintenance of osmotic imbalance is considered a protective mechanism to alleviate the oxidative stress under salt stress in plants [58]. In many studies, it has been reported that the inoculation of *Bacillus* diminished the ROS generation and reduced the MDA content under salt stress [3,11,44]. In other studies, *B. subtilis* also restricted the ROS generation and declined the MDA content in cadmium-spiked *G. max* [59] and drought-affected *T. aestivum* [15].

To defend against the ROS-induced oxidative damages under salinity, plants rely on activating ROS-scavenging machinery, including non-enzymatic and enzymatic antioxidant enzymes. The non-enzymatic antioxidants, AsA and GSH, are highly active in counteracting ROS generation and maintaining homeostasis, protecting the cellular organelles against oxidative disruptions [60]. Ascorbate (AsA) interrupts the chain reaction of ROS generation by donating electrons to APX under stresses and is also involved in the biosynthesis of phytohormones. At the same time, GSH is a low molecular thiol tripeptide that degrades H_2_O_2_ in the catalyzing reaction with the help of GPX [7]; thus, the alone or combined actions of AsA and GSH play an important role in stress-sensing and adaptation. Here, salt stress caused a declination of AsA and the AsA/DHA ratio as a result of increased DHA (the oxidized form of AsA); thus, the salinity increased the ROS accumulation in the soybean plants, which is consistent with the earlier findings [5,46]. The AsA-metabolizing enzyme, APX, was increased, but the MDHAR and DHAR activities were reduced in the salt stressed soybean. In the AsA-GSH cycle, APX converts the H_2_O_2_ to H_2_O by adopting an electron from the AsA, turning it into monodehydroascorbate (MDHA). A further regeneration of AsA from the MDHA was occurred with the help of MDHAR [4]. Similar responses of AsA, AsA/DHA, and AsA-recycling enzymes (APX, MDHAR, DHAR) were reported in salt affected *B. napus* [61]. A sharp increment of GSSG was responsible for reducing the GSH content and GSH/GSSG ratio in soybean under salt stress. Here, by using GSH, the DHA turned into AsA, which resulted in the production of GSSG. Finally, the GSH was regenerated from the GSSG, obtaining an electron from the NADPH by the activity of GR and restoring redox homeostasis [7,62]. Salt affected *Corchorus olitorius* plants showed a reduced accumulation in GSH and the GSH/GSSG ratio with an increase in GSSG [5], which is in congruence with the present findings; however, an improved GR activity recovered the GSH and sustained the GSH/GSSG ratio to regulate ROS-scavenging to alleviate oxidative stresses. A similar enhancement of the GR activity and GSH content were reported by Parvin et al. [8] in salt-stressed *Solanum lycopersicum* plants. Moreover, an inoculation of *B. subtilis* further upregulated the AsA and GSH contents and reduced the DHA and GSSG contents, thereby increasing the AsA/DHA and GSH/GSSG ratios in soybean, which is an indication of more efficient ROS detoxification under salt stress. Previous reports have also demonstrated that *Bacillus* inoculation upgraded the AsA and GSH production in *T. aestivum* [15] and *Abelmoschus esculentus* [63].

The antioxidant enzymes, SOD, CAT, POD, GST, and GPX are also engaged in detoxifying ROS to maintain redox homeostasis. The enzyme SOD activates its activity as the frontline of defense through the conversion of O_2_^•−^ into H_2_O_2_ and the further quenching of H_2_O_2_ into H_2_O by the activity of CAT [64]. The activities of SOD and CAT were decreased in the salt affected soybean plants, which might have been due to the increase in H_2_O_2_ responsible for inducing oxidative stress. A similar reduction in the activities of SOD [65] and CAT [66] was reported under salt stress. Moreover, the enzymes GPX and GST are also involved in detoxifying ROS by using GSH as a substrate. The GPX, as a member of the non-heme POD family, utilizes the GSH and thioredoxins to scavenge H_2_O_2_ and other lipid oxidizing compounds. In contrast, other xenobiotic compounds are also conjugated with the aid of GSH and electrophilic substances [4]. The activities of POD, GST, and GPX were increased in a concentration-dependent manner in soybean under salt stress, indicating ROS detoxification; however, the *Bacillus* accelerated the antioxidant enzyme activities under different abiotic stress [3,59,67,68]. The previous finding of PGPR on antioxidant enzyme activities supports the current study with *B. subtilis* inoculation. It could be stated that beneficial microbes can improve the antioxidant defense system by enhancing different metabolic activities under salt stress.

Methylglyoxal is an α-oxoaldehyde compound that is highly reactive and cytotoxic, produced intracellularly in a trace amount under normal conditions but overproduced under stressed conditions [7]. When the soybean plants were exposed to salt stress, a notable increase in MG was found due to the reduction in MG detoxifying enzyme (Gly I and Gly II) activities. The decreased activities of Gly I and Gly II were also reported in the previous investigations on salt affected plants [5,61]. In the glyoxalase system, MG detoxification takes place in a two-step reaction. First, Gly I utilizes GSH to convert the MG into SLG, and in the second step, the conversion of SLG to D-lactic acid and the rejuvenation of GSH occurs by the activity of the Gly II enzyme [7]. The GSH acts as a cofactor of the glyoxalase system that mediates the reactions and plays a crucial role in MG detoxification. Upon the inoculation of the *B. subtilis*, the GSH production was increased, thus, enhancing the Gly I and Gly II activities in soybean plants, and ultimately diminished the over-accumulated MG under salt stress.

As a whole, the *B. subtilis* inoculation augmented the AsA and GSH contents with reduced DHA and GSSG contents being observed, enhancing the AsA/DHA and GSH/GSSG ratios. The accelerated activities of the APX, MDHAR, DHAR, GR, SOD, CAT, POD, GST, and GPX enzymes restored the antioxidant defense system in the salt stressed soybean. Consequently, the *B. subtilis* inoculation enhanced the RWC, decreased the Pro content, and accelerated ROS-scavenging to mitigate the oxidative stress-induced damages. Further restoration of ion homeostasis was also seen in the *B. subtilis*-inoculated plants under salt stress. Moreover, the *B. subtilis* inoculated plants showed an upregulation of the Gly I and Gly II activities that reduced the MG content and associated adversities in the plants under salt stress.

## 5. Conclusions

The findings of the present study demonstrate the protective role of *B. subtilis* in preventing the damaging effects of salt stress and salt-induced oxidative stress in soybean, by improving different morpho-physiological and biochemical parameters. The inoculation of *B. subtilis* enhanced the activities of both antioxidant enzymes, viz., APX, MDHAR, DHAR, GR, SOD, CAT, POD, GST, and GPX, as well as glyoxalase enzymes, viz., Gly I and Gly II. Consequently, it helped in accelerating the ROS-scavenging along with the diminished MG toxicity to mitigate the oxidative stress-induced damages in soybean plants. Moreover, ion homeostasis was maintained in the *B. subtilis*-inoculated plants through the upregulated accumulation of K^+^ and Ca^2+^ under salt stress. Furthermore, the plant growth, biomass, photosynthetic pigments, and water status were uplifted with *B. subtilis* inoculation in the salt-induced soybean plants, and interestingly, the soil inoculation performed better in improving the defensive mechanisms of soybean in most cases. Therefore, it can be stated that *B. subtilis* is an efficient tool to mitigate salt-induced oxidative stress through the modulation of ROS-scavenging, antioxidant defense system, and glyoxalase system to boost the growth and development of plants under salinity. However, the precise role of *B. subtilis* and its complex association with the diverse biochemical processes and the related signaling pathways for salt stress tolerance has not been studied thoroughly yet; therefore, this warrants further investigations.

## Figures and Tables

**Table 1:** Effect of *Bacillus subtilis* inoculation on growth attributes of soybean (*Glycine max* cv. BARI Soybean-5) under different levels of NaCl. Values in a column are mean ± SD. Different letters on each bar indicate significant differences among the treatment after applying a Tukey’s HSD test at *p* ≤ 0.05.

Salt Concentration (NaCl)	Bacterial Inoculation	Shoot Length (cm)	Root Length (cm)	Stem Diameter (mm)	Root FW (g Plant^−1^)	Shoot FW (g Plant^−1^)	Root DW (mg Plant^−1^)	Shoot DW (mg Plant^−1^)
0 mM	Control	36.95 ± 0.50 ab	9.36 ± 0.26 ab	1.87 ± 0.05 ab	0.29 ± 0.01 a	3.79 ± 0.11 ab	55.4 ± 1.61 a	735.8 ± 13.42 a
	Seed inoculation	39.52 ± 1.6 a	9.64 ± 0.76 ab	1.95 ± 0.14 a	0.31 ± 0.02 a	4.10 ± 0.18 a	56.4 ± 2.78 a	770.4 ± 39.14 a
	Soil inoculation	39.80 ± 1.53 a	10.43 ± 0.82 a	1.95 ± 0.06 a	0.31 ± 0.01 a	3.99 ± 0.07 ab	53.2 ± 1.97 a	770.0 ± 25.43 a
50 mM	Control	31.94 ± 2.94 bcd	7.9 ± 0.22 cde	1.71 ± 0.10 abc	0.17 ± 0.01 de	2.76 ± 0.17 e	30.7 ± 0.38 cd	379.2 ± 17.48 def
	Seed inoculation	35.35 ± 1.55 abc	8.67 ± 0.50 bcd	1.99 ± 0.13 a	0.20 ± 0.012 bc	3.19 ± 0.21 d	35.0 ± 2.14 c	478.3 ± 24.80 bc
	Soil inoculation	36.25 ± 2.75 ab	9.76 ± 0.86 ab	1.83 ± 0.03 abc	0.21 ± 0.01 b	3.62 ± 0.08 bc	41.0 ± 0.90 b	542.2 ± 48.00 b
100 mM	Control	28.23 ± 2.01 de	7.22 ± 0.45 de	1.56 ±0.01 c	0.14 ± 0.01 ef	2.06 ± 0.14 f	23.0 ± 1.77 ef	335.0 ± 13.26 efg
	Seed inoculation	34.98 ± 1.10 abc	8.31 ± 0.60 bcd	1.81 ± 0.03 abc	0.19 ± 0.01 bcd	2.90 ± 0.15 de	32.4 ± 0.56 cd	385.6 ± 20.15 de
	Soil inoculation	34.46 ± 2.33 abc	9.14 ± 0.77 abc	1.82 ± 0.03 abc	0.21 ± 0.01 bc	3.26 ± 0.22 cd	34.8 ± 3.02 c	446.3 ± 23.11 cd
150 mM	Control	25.75 ± 1.23 e	6.63 ± 0.43 e	1.23 ± 0.24 d	0.12 ± 0.01 f	1.01 ± 0.05 g	22.1 ± 0.45 f	196.6 ± 8.83 h
	Seed inoculation	30.47 ± 1.55 cde	7.75 ± 0.27 cde	1.60 ± 0.06 bc	0.18 ± 0.01 cd	1.74 ± 0.08 f	27.8 ± 0.58 de	294.5 ± 17.15 g
	Soil inoculation	30.56 ± 1.52 cde	8.6 ± 0.20 bcd	1.54 ± 0.07 c	0.16 ± 0.01 de	1.69 ± 0.04 f	31.0 ± 2.05 cd	307.8 ± 27.30 fg

## Data Availability

All data are available in this manuscript.
